# Barriers and facilitators for participation in workplace health promotion programs: results from peer-to-peer interviews among employees

**DOI:** 10.1007/s00420-022-01930-z

**Published:** 2022-10-28

**Authors:** Denise J. M. Smit, Karin I. Proper, Josephine A. Engels, Jennifer M. D. Campmans, Sandra H. van Oostrom

**Affiliations:** 1 Center for Nutrition, Prevention and Health Services, National Institute for Public Health and the Environment, Bilthoven, The Netherlands; 2 Department of Public and Occupational Health, Amsterdam UMC, Vrije Universiteit Amsterdam, Amsterdam Public Health Research Institute, Amsterdam, The Netherlands; 3 HAN University of Applied Sciences, Occupation and Health Research Group, Nijmegen, The Netherlands

**Keywords:** Employees, Integrated workplace health promotion, Peer-to-peer interviews, Participation, Consolidated Framework for Implementation Research, Social ecological model

## Abstract

**Objective:**

Workplace health promotion programs (WHPPs) have shown to be effective in improving lifestyle behaviors of employees. Despite potential benefits for employees, participation rates are generally low. The aim of this study was to gain deeper insight into barriers and facilitators for participation in WHPPs prior to implementation according to employees.

**Methods:**

Peer-to-peer interviewing, a method derived from citizen science, was used to actively involve employees in the data collection. Employees working in the cleaning-, ICT- and facility-sector were trained to interview their co-workers. Interviews were recorded and transcribed verbatim. Thematic analysis was performed using the Consolidated Framework for Implementation Research (CFIR), complemented with the constructs ‘interpersonal factors’ and ‘intrapersonal factors’ from the social ecological model. Data were coded deductively and inductively, and rated by two researchers independently.

**Results:**

Fourteen peer-interviewers conducted 62 peer-to-peer interviews. Main barriers for participation in WHPPs were an unsupportive organizational culture where lifestyle is not a common topic and programs that are not tailored to their needs. Support from peers and supervisors were facilitators. The availability of organizational resources, such as facilities and financial compensation, support participation.

**Conclusions:**

To enhance participation of employees in WHPPs it is recommended to take into account the barriers and facilitators identified in this study. For instance, employees should be involved in the development and implementation of WHPPS by the employer and their needs and available resources should be taken into account. This may lead to more successful implementation and higher participation rates in future WHPPs.

## Introduction

The workplace is an ideal setting to promote a healthy lifestyle, among others as it can reach a large group of adults and because of existing infrastructures for interventions (Goldgruber and Ahrens 2009; Robroek et al. 2009). Employers can implement health promoting activities on top of their legal responsibility to secure sustainable working conditions for their employees. Effectiveness of workplace health promotion (WHP) on several targeted lifestyle behaviors such as diet, physical activity, and psychological health is demonstrated in multiple studies (Carolan et al. 2017; Maes et al. 2012; Proper and van Oostrom 2019; Verweij et al. 2011). Employees can benefit in terms of improved lifestyle, and eventually improved health. WHP programs (WHPPs) have proven to be effective in weight loss, increased psychological wellbeing and perceived health of employees (Carolan et al. 2017; Rongen et al. 2013; Verweij et al. 2011). Despite the potential benefits for employees, reported participation rates of WHPPs vary greatly. Robroek et al. found that participation levels varied from 10 to 64%, with a median of 33% (Robroek et al. 2009). Low levels of participation can negatively affect the effectiveness and cost-effectiveness of WHPPs and limit their reach and impact (Linnan et al. 2001; Robroek et al. 2009, 2021). Both adequate implementation and high levels of participation are crucial factors for the effectiveness of a WHPP (Durlak and DuPre 2008; Linnan et al. 2001; Robroek et al. 2009, 2021).

Multiple barriers and facilitators for participation in existing WHPPs have been reported (Robroek et al. 2009, 2012; Rongen et al. 2014; Schubin et al. 2020; Sigblad et al. 2020). Earlier barriers identified were related to the employees’ responsibility of a healthy lifestyle, a lack of time and the preference to improve lifestyle in one’s own time (Robroek et al. 2012; Rongen et al. 2014; Schubin et al. 2020; Sigblad et al. 2020). Factors that had a positive impact on participation were a program that focused on multiple lifestyle themes and a multicomponent program, e.g. a program with both an individual- and an environmental approach (Robroek et al. 2009). A positive attitude of employees towards WHPPs and high levels of support were associated with a positive intention towards participation in WHPPs (Rongen et al. 2014; Sigblad et al. 2020). Recent qualitative studies towards barriers and facilitators for participation were not directly from an employees’ perspective, but for example from a managers’ or occupational physicians’ view (Schubin et al. 2020; Sigblad et al. 2020). This implies that there is a need to expand the body of knowledge about barriers and facilitators for participation from an employees’ perspective.

Adequate implementation of WHPPs can positively influence participation rates. Implementation can be improved when (1) barriers and facilitators are identified during the pre-implementation phase and (2) when employees are actively involved in the implementation and design of the program (Henning et al. 2009; Hunt et al. 2000; Person et al. 2010; Project 2013; Robroek et al. 2012, 2021; Sigblad et al. 2020; Tonnon et al. 2014; Varsi et al. 2015; WHO 2008). In practice, barriers and facilitators for participation in a WHPP are often collected after program implementation (Robroek et al. 2012; Schubin et al. 2020; Sigblad et al. 2020). Preferably, barriers are known prior to implementation, so strategies to overcome these barriers can be developed beforehand. To further improve implementation, a citizen science method can be applied to actively involve employees (Den Broeder et al. 2018; Project 2013; Tsui and Franzosa 2018). This engagement can be created on various levels, for instance, participants can provide data collection (Den Broeder et al. 2018).

This study was conducted during the development of an integrated WHPP in which a good example of a successful integrated WHPP, the Lombardy WHP Network (LWHPN), was tailored to the Dutch context (Smit et al. 2022). The LWHPN is recognized as a good practice in the occupational setting in the European Joint Action CHRODIS because of its integrated approach and successful implementation (CHRODIS 2014; Coppola et al. 2016; Public Health Best Practice Portal 2020). Integrated WHPPs target multiple lifestyle themes at both the individual and organizational level (Booth et al. 2001). Previously reported barriers and facilitators were often found for programs that focused on one specific lifestyle theme and not for integrated WHPPs (Robroek et al. 2009; Schubin et al. 2020). The aim of this study was to gain deeper insight into barriers and facilitators for participation in WHPPs according to employees prior to the implementation of an integrated WHPP. This involves factors at both the organizational and individual level that may facilitate or hamper participation in WHPPs according to employees. Insight into these barriers and facilitators might help to increase participation of employees in the integrated WHPP and future WHPPs.

## Methods

### Study design

For this study we used a qualitative design, employing peer-to-peer interviews. Peer-to-peer interviewing is a method derived from citizen science, which means that participants actively engage in carrying out research (Den Broeder et al. 2018; Tsui and Franzosa 2018). Peer-to-peer interviews have several benefits, such as efficient data collection and participants are considered to respond more genuinely to their peers, which leads to less socially desirable answers (Byrne et al. 2015; Devotta et al. 2016; Tsui and Franzosa 2018). Data were collected between October 2020 and January 2021.

The Center for Clinical Expertise of the Dutch National Institute of Public Health and the Environment classified the study as exempt from ethical review as it did not meet the criteria of the Medical Research Involving Human Subjects Acts. The center approved the study protocol (study number VPZ-458). Informed consent was obtained from all interviewees and the peer-interviewers.

### The integrated WHPP

The integrated WHPP to be implemented exists of (1) a catalogue with health promoting activities on multiple levels (individual and organizational) and multiple lifestyle themes (physical activity, nutrition, relaxation, smoking, work-life balance, alcohol consumption, stress and sleep) and (2) an implementation plan to support successful implementation (Smit et al. 2022). The choice for these lifestyle themes was based on relevance according to both employers and employees. Examples of activities in the catalogue on the individual level are dissemination of information (e.g. about the importance of a healthy diet, a healthy work-life balance and smoking cessation), deploying exercise challenges or providing tools to monitor lifestyle (activity tracker, nutrition app). Examples of activities on the organizational level are adjustments to the working environment (offering healthy foods in the company restaurant, availability of sit-stand desks) or to the social environment (managers as role models, small social events, such as coffee breaks). Potential barriers and facilitators for implementation and participation were used to develop the implementation plan. A working group within the organization, consisting of employees, HR professionals, managers, and prevention workers will select and implement activities from the catalogue according to the criteria of the integrated approach. This way, both employers and employees are involved, and the integrated WHPP can be adapted to local needs and available resources.

### Recruitment

This study was embedded in a larger study in which an integrated WHPP will be developed, implemented and evaluated (Smit et al. 2022). Organizations that will participate in the cluster randomized controlled trial (c-RCT) to evaluate the integrated WHPP were recruited trough the network of the research team, co-workers and branch specific networks and platforms. Organizations could participate in the c-RCT when they had not yet implemented an integrated WHPP (i.e. implemented activities on both the individual and organizational level within multiple lifestyle themes). Organizations were not systematically asked for their motivation to participate in the c-RCT. However, conversations with organizations revealed that it involved contributing to the health and sustainable employability of employees.

Peer-interviewers and interviewees for the current qualitative study were recruited within two of the organizations that agreed to participate in the c-RCT. A cleaning company and two departments of a University of Applied Sciences, the ICT- and a facility-department. Peer-interviewers were recruited by (1) supervisors within the organization who informed employees about the study and asked them to participate as a peer-interviewer, or (2) a short presentation by one of the researchers (DS) on the aim and process of the peer-interviewing. Afterwards employees could sign up as a peer-interviewer. All employees who spoke and understood Dutch were eligible to participate as a peer-interviewer, with the exception of employees in a management position. None of the peer-interviewers had prior interview experience. Peer-interviewers were asked to interview five co-workers from their department who differed in age, sex, and job function, to create a heterogenic study population.

### Data collection

All peer-interviewers followed an online training of 2 hours, provided by the researchers. In the training, they were educated on how to conduct an interview with a co-worker, were informed about how to obtain informed consent from their co-workers, practiced their interview skills with other peer-interviewers and received feedback from the researchers.

The interviews were semi-structured and included three main questions: (1) about what employees think about when it comes to lifestyle; (2) about the current offer of WHPPs by their employer and whether and why they would participate or not and; (3) about the way they would like to be informed about WHPPs within their organization. To assist the peer-interviewers, they received cards with interview instructions, information about the study, main questions, sub-questions per main question and tips for further follow-up questions. Furthermore, they were instructed to listen carefully to their co-workers and adapt and personalize the follow-up questions when deemed appropriate. Additionally, age, sex, working hours, years of working at the organization and job type were asked. The main and sub-questions are depicted in Table 1. One-on-one interviews were performed at the workplace and could be face-to-face or online. This depended on the work situation of the peer-interviewers, since working from home was part of the COVID-19 restrictions at the time of this study. Interviews were audio or online recorded.

**Table 1:** Main and sub-questions of the semi-structured interviews

	Main questions	Sub-questions
1	When you think about lifestyle, what do you think about?	What would you like to improve, regarding your lifestyle?
		How could your employer help you to improve your lifestyle?
2	Does your employer organize activities to improve your lifestyle?	Did you participate in such an activity?
		Under what circumstances would you participate in such an activity?
		Under what circumstances would you not participate in such an activity?
3	How would you like to be informed about activities at work to improve your lifestyle?	

### Theoretical framework for qualitative analysis

The Consolidated Framework for Implementation Research (CFIR) was used as framework for the thematic analysis, complemented with two constructs of a social ecological model (Braun and Clarke 2006; Damschroder et al. 2009; Linnan et al. 2001). The CFIR is an overarching framework to guide implementation research in which multiple implementation frameworks are integrated (Damschroder et al. 2009). The CFIR was chosen for this study because of its comprehensiveness and fit in the implementation of WHPP (Lash et al. 2011; Robins et al. 2013; Varsi et al. 2015). The framework consists of five domains: (1) intervention characteristics, (2) outer setting, (3) inner setting, (4) characteristics of individuals, and (5) process. ‘Intervention characteristics’ contains key attributes of the WHPP, ‘outer setting’ addresses the external environment whereas the ‘inner setting’ describes the situation within the organization. The domain ‘characteristics of individuals’ is associated with the actions and behaviors of the involved individuals, in this case, the employees. The domain ‘process’ involves implementation strategies. Constructs within these domains are expected to influence implementation. Hence, the CFIR can assist in identifying barriers and facilitators for implementation of a WHPP (Damschroder et al. 2009; Kirk et al. 2016).

The CFIR is originally applied from the implementers’ perspective. Since the focus of this study is on the barriers and facilitators of participation according to employees, definitions of some constructs had to be adapted. The domain characteristics of individuals originally addressed the characteristics of implementers. In this study it addresses characteristics of employees, i.e. the users of the program. The outer setting of the CFIR also included the construct patient needs and resources, for the purpose of this study we transferred this construct to the domain characteristics of individuals. Additionally, needs and resources of employees were included as two separate constructs. The construct peer pressure, from the domain outer setting, was adjusted to peer support in the inner setting. The definition of the construct leadership engagement was adapted, so it focused on the role of supervisors in motivating and stimulating employees to participate. The construct available resources originally focused on the level of resources made available for implementation. This was replaced within the construct organizational resources, which targets the facilities and time provided by the organization to enable participation in WHPPs for employees. The construct knowledge and beliefs about the intervention is split into two separate constructs, i.e. knowledge about and familiarity with the intervention and beliefs about the intervention. Furthermore, the domain characteristics of individuals was extended with two constructs, the ‘interpersonal factors’ and ‘intrapersonal factors’, of the social ecological model (Linnan et al. 2001). Social ecological models are a useful tool to explain behavior of an individual, for instance participation in a WHPP (Linnan et al. 2001).

### Analysis

**Fig. 1:**
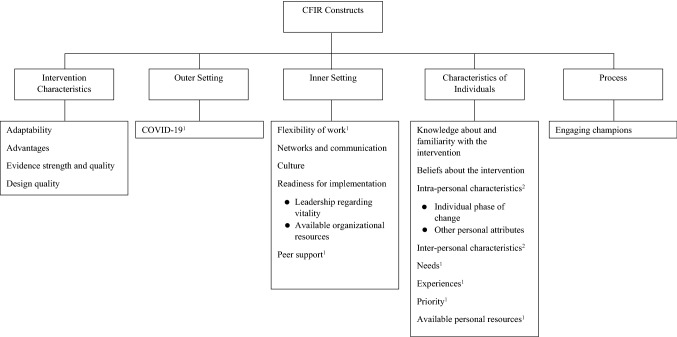
Overview of the constructs, mainly based on the CFIR

Interviews were transcribed verbatim and, after familiarization, analyzed by two researchers (DS, JC). Two steps of the analysis of qualitative data according to the CFIR were followed: (1) thematic coding and (2) rating. In the first step, the existing codebook of the CFIR with the additional constructs of the social ecological model was used to code the data (Damschroder et al. 2009; Linnan et al. 2001). A hybrid approach was applied, which allows for both inductive and deductive coding (Fereday and Muir-Cochrane 2006). Additional codes that emerged from the data were added to the codebook (inductive). In total, 21 constructs of the CFIR and two constructs of the social ecological model were used and seven constructs were added (Fig. 1). The MAXQDA 2020 software was used for the thematic coding process. In total, six interviews were double coded independently by the two researchers, afterwards the interviews were compared and discussed until consensus was reached. The remaining interviews were divided under the researchers, coded, and checked by the other researcher. Discrepancies were discussed until consensus was reached. A third researcher (SO) was consulted in case of disagreement. Due to the hybrid approach, the codebook was continuously enriched with new codes, prior coded interviews were recoded if necessary.

In the second step, the constructs were rated to establish (1) the valence of a construct, i.e. the positive or negative influence of the construct on participation and (2) the strength of this influence. Constructs could also be rated to have a neutral or mixed influence on participation (Table 2). Ratings were assigned based on the qualitative data from individual transcripts (Consolidated Framework for Implementation Research Qualitative Data 2022). The rating criteria were slightly adapted from those reported by the CFIR developers since the CFIR is not applied from the implementers’ perspective in this study (Damschroder and Lowery 2013). Instead of impeding or facilitating factors for implementation, we assessed impeding or facilitating factors for participation. The coded segments were double rated independently by two researchers (DS, JC). Afterwards, ratings were compared and discussed until consensus was reached, in case of disagreement, a third researcher (SO) was consulted.

**Table 2:** Rating criteria applied in the rating step

− 2	The construct is an impeding influence for participation in WHPPs by employees. The majority of employees describe explicit examples of how the construct manifests itself in a negative way
− 1	The construct is an impeding influence for participation in WHPPs by employees. Employees make general statements about the construct manifesting in a negative way but without concrete examples
0	A construct has neutral influence if it appears to have a neutral effect, i.e. no obvious positive or negative influence
X	The construct can have a mixed rating if the comments are both positive and negative
+ 1	The construct is a facilitating influence for participation in WHPPs by employees. Employees make general statements about the construct manifesting in a positive way but without concrete examples
+ 2	The construct is a facilitating influence for participation in WHPPs by employees. The majority of employees describe explicit examples of how the key or all aspects of a construct manifests itself in a positive way

## Results

### Characteristics of the participants

Fourteen peer-interviewers were trained and conducted 1–6 interviews each. Three peer-interviewers worked for a cleaning company, seven worked at a facility department and four worked at an ICT department. In total, the peer-interviewers conducted 62 peer-to-peer interviews, which lasted between 3 and 25 min. Ten interviewed employees worked for a cleaning company, 34 worked at a facility department, and 18 worked at an ICT department. Characteristics of the 62 interviewed employees are further specified in Table 3.

**Table 3:** Characteristics of employees who participated in an interview (*n* = 62*)

	Total	Cleaning company	Facility department	ICT department
Age in years (mean, SD)	49.5 (9.5)	45.6 (8.3)	50.1 (10.0)	50.1 (8.4)
Sex (m/f)	31/31	1/9	16/18	14/4
Working hours per week by contract (mean, SD)	32.6 (8.0)	32.2 (10.5)	31.7 (8.2)	34.9 (4.4)
Years of working at the organization (mean, SD)	9.7 (7.5)	11.4 (3.0)	7 (6.6)	13.9 (8.2)
Summary of job types	N.A	Cleaner, allround employee	Concierge, receptionist, campus store sales representative, security guard, process coordinator	Administrator IT, employee service desk, system administrator

*Descriptive data from eight employees were not complete

### Barriers and facilitators

The findings are described based on the rating of the constructs. Details about the rating are displayed in Table 4.

**Table 4:** Rating of the constructs

Construct	Rating
**Intervention characteristics**	
Adaptability	+ 1
Advantages	+ 2
Evidence strength and quality	+ 1
Design quality	X
**Outer setting**	
External policies	0
COVID-19	− 1
**Inner setting**	
Flexibility of work	− 2
Networks and communication	+ 1
Culture	− 2
Tension for change	0
Compatibility	0
Relative priority	0
Goals and feedback	0
Leadership regarding vitality	+ 1
Available organizational resources	X
Peer support	+ 1
**Characteristics of individuals**	
Knowledge about and familiarity with the intervention	− 2
Beliefs about the intervention	X
Self-efficacy	0
Individual phase of change	X
Other personal attributes	− 1
Interpersonal characteristics	− 1
Individual identification with the organization	0
Needs	− 1
Experiences	+ 2
Priority	X
Available personal resources	− 1
**Process**	
Stakeholders	0
Champions	+ 1

#### Intervention characteristics

Within this domain the constructs ‘advantages’ (+ 2), ‘evidence strength and quality’ (+ 1), and ‘adaptability’ (+ 1) were facilitators for participation. Employees are more likely to participate in WHPPs when they are aware of the advantages of a program, in terms of health benefits including both physical- and social-health effects. Moreover, personal goals that can be achieved through participation in WHPPs are seen as advantages and might, therefore, facilitate participation. When there is evidence that a program can lead to increased health, employees indicate to be more willing to participate. This can be achieved by informing employees about the potential of proven effects. Employees also indicate that they would be more inclined to participate when programs are adapted to their age, their daily working schedule, or when there is sufficient choice in locations or types of sports: “*Well you know what would help me, and I believe [the organization] also offers opportunities for that, is for example fitness. But then tailored to my body or my age, or to my goals*” (Facility department, male, age 49).

‘Design quality’ (x) can both facilitate and hinder participation. Employees mention that a high quality WHPP, i.e. an evidence- or practice-based WHPP, can positively influence participation. The experienced quality can be improved by involvement of professionals or students. A mandatory program is a barrier for participation according to employees, whereas a program free of charge will support participation.

#### Outer setting

The *‘*COVID-19 pandemic’ (− 1) appeared to be a barrier for participation in WHPPs. Employees do not want to be at risk of becoming infected when they participate in a WHPP: “*Peer-interviewer: And under which circumstances would you no longer participate in such activities? Employee: Well, that answer is actually quite simple, because of COVID-19. Because were it not for COVID-19, I would just participate*”. (ICT Department, male, age 32).

#### Inner setting

The constructs ‘peer support’ (+ 1), ‘leadership regarding vitality’ (+ 1) and ‘networks and communication’ (+ 1), were identified as facilitators in this domain. Peer support can trigger employees to participate in WHPPs, as it brings an additional social component and co-workers can motivate each other. On the other hand, a small group of employees feels no need to engage in lifestyle related activities with their co-workers. For example, because they prefer to exercise on their own. With regard to the construct leadership, employees shared that information about the importance and possibilities of WHPPs provided by supervisors or managers can support their participation. This is also the case for supervisors who actively motivate and support their employees to participate: “*And when your supervisor indicates that it [WHPP] is good for you. [⋯] Then you might also literally get people moving who otherwise might not have signed up for something of their own accord*”. (Facility department, female, age 25).

Employees indicated that when communication about the WHP possibilities within the organization is clear and sufficient, there possibly is a lot of enthusiasm for it among their selves and co-workers: “*Peer-interviewer: When would you participate in such an activity though? Employee: If I were informed a little bit more. What the concrete possibilities are within [the organization]*”. (Facility department, male, age 54). Additionally, employees mentioned they would like to be informed about WHPPs via presentations, information markets or other visible manners, posters, by phone, intranet, social media, newsletters, a personal approach and e-mail. However, an overload of information should be avoided, as this can lead to ambiguities or a lack of interest. Employees prefer active distribution of information, since it is not likely that they are going to look for information about WHPPs on their own initiative.

A lack of ‘flexibility of work’ (− 2) and an unsupportive ‘organizational culture’ (− 2) were identified as barriers within the inner setting domain. Employees who are not able to leave their workplace during work time or have no flexibility to start or stop working later or sooner than scheduled, stated that it hampers their participation in WHPPs: “*I know that Tai Chi, yoga and office yoga, or the like, are organized during lunch breaks, and walks too, but it’s just very difficult to leave this workplace. You can’t leave the reception unoccupied. [⋯] Or you would have to arrange replacements, but I think that is a bit difficult. Maybe I do not have enough of a nine to five mentality and feel too much responsible to leave my workplace for something like that*”. (Facility department, female, age 49). Employees indicated that they would not participate in WHPPs when faced with an unsupportive organizational culture. For example when participation in WHPPs, especially during working hours, is not commonly accepted by co-workers: “*Peer-interviewer: Look, in the past there also have been activities that were organized so to say, during the day, do you experience any obstacles to participate in such activities because it is during working hours? Because that's where you end up, isn’t it? Employee: Yes, that’s true, [⋯] that restraint is still there. [⋯] it’s not yet such a widely accepted, given*”. (Facility department, male, age 42). Additionally, in a culture where employees see their health and lifestyle as something private and not as something work-related, participation is hampered.

The construct ‘available organizational resources’ (x) had a mixed rating. The availability of a financial compensation, for example for a gym or other sport will support participation. In contrast, high prices for healthy food in the company restaurant will hamper participation. According to employees, they are more likely to participate when the location of a WHPP is easy to reach. Hence, facilities such as a gym at the workplace are facilitators for participation with the lack of such facilities being a barrier. Another factor is time, the possibility to participate during working hours can be a facilitator, since a lack of time after working hours is a frequent barrier: “*Peer-interviewer: What could the employer really do to make you participate? [⋯]. Employee: Maybe if you are allowed to participate during working hours? Then you are more inclined to participate. Besides that I wouldn’t know*”. (ICT department, male, age 47).

#### Characteristics of individuals

The construct ‘experiences’ (+ 2) was the only facilitator within the domain characteristics of individuals. Employees suggest that positive experiences with a WHPP, such as feeling healthier or having a good time, would be a reason to participate in other WHPPs as well.

‘Knowledge about and familiarity with the intervention’ (− 2), ‘other personal attributes’ (− 1), ‘personal resources’ (− 1), ‘interpersonal characteristics’ (− 1), and ‘needs’ (− 1) were identified as barriers in this domain. With regard to knowledge and familiarity, it was emphasized that if employees were not aware of a program or when programs are unclear, they would not participate: “*Look, if you don’t know about the existence of WHP activities, then you’re not going to use them either*”. (ICT Department, male, age 38). Personal attributes such as injuries or a lack of energy, and a lack of personal resources such as time and financial resources also hinder participation. Family and friends, e.g. interpersonal characteristics, can motivate employees. However, the time and energy that is spent on a busy household hamper participation in WHPPs outside working hours. Also, employees indicate that they prioritize time spent with family over participation in WHPPs. Employees mention that they will not participate when a program does not fit their needs or when they do not enjoy it: “*[⋯] When I wouldn’t participate in a challenge or something, if it isn’t really in my field of interests, yes that would be my answer. Actually it’s very simple*”. (ICT department, male, age unknown). Employees who are already engaged in a healthy lifestyle, employees who do not recognize that their current lifestyle should be improved and employees who see their work as physical exercise, do not feel any need for participation in WHPPs: “*Peer-interviewer: What would make you participate in such an activity? Employee: When it involves sports and exercise, I would not participate, because I get enough of those already. I mean through cleaning*” (Cleaning company, female, age 26).

The constructs *‘*beliefs about the intervention’, ‘priority’ and ‘individual phase of change’ had a mixed rating (x) within this domain. Employees who believe that improving lifestyle is something you have to do yourself, and not something that your employer should facilitate, are less inclined to participate in WHPPs: “*Peer-interviewer: How can your employer help you to improve your lifestyle? Employee: Well I don’t think that he [employer] can improve it [my lifestyle] that much, because it’s something that I have to do myself*”.* (*Cleaning company, female, age 50). The same goes for employees who indicate that they do not know how their employer could facilitate a healthy lifestyle of employees. On the other hand, employees with a positive attitude towards and belief in WHPPs, are more open to participation. When employees see the benefits and necessity of a WHPP, the construct priority is a facilitator. On the contrary, if employees are already actively engaged in a healthy lifestyle in their private time, their priority will not be to participate in WHPPs. Besides, most employees said to prioritize their work over lifestyle at the workplace, making priority a barrier as well. When employees are aware of the importance of a healthy lifestyle, it is more likely that they will participate. In contrast, if an employee is not aware of the importance of a healthy lifestyle, or when they do not recognize that there is a problem with their lifestyle, their individual phase of change hampers participation in WHPPs.

#### Process

The engagement of ‘champions’ (+ 1) was mentioned as a facilitator for participation. Enthusiastic employees might convince co-workers to participate and they can serve as a role model.

## Discussion

### Main findings

From the perspective of employees, positive experiences and knowledge about advantages of participation were important facilitators for participation. The most important barriers for participation in WHPPs were an unsupportive organizational culture, a lack of knowledge about WHPPs and various individual characteristics, such as a lack of personal resources. Organizational resources could act as both a facilitator and a barrier for participation.

### Comparison with literature

Multiple other studies that identified barriers and/or facilitators according to employees were found with some similar findings (Nöhammer et al. 2010, 2013; Person et al. 2010; Robroek et al. 2012; Rongen et al. 2014). A perceived healthy lifestyle was a frequently mentioned barrier (Robroek et al. 2012; Rongen et al. 2014), a barrier that also came forward in our study. This might imply that employees are indeed already engaged in a healthy lifestyle or that they do not recognize that their lifestyle needs improvement. Misperceptions about health and lifestyle are a known barrier for adapting lifestyle behaviors in general, possibly due to a lack of knowledge or awareness (Tonnon et al. 2014). For instance, there often is a lack of knowledge about the different health effects of exercising in leisure time and occupational physical activity (OPA) (Holtermann et al. 2018). Literature shows that OPA can negatively affect health, whereas exercising in leisure time can benefit health (Holtermann et al. 2018). We found that employees with physically demanding jobs indicate that they do not need to exercise, because of the high OPA. This finding might imply a lack of knowledge about lifestyle and health, specifically for physical activity. Or it might suggest that employees with physically demanding jobs experience a lack of energy due to high OPA, which can be a barrier for participation in physical activity in leisure time. Other reasons for non-participation, in line with our findings, were not knowing about a WHPP, a preference to keep work and private life separate, inconvenient locations and a lack of time (Nöhammer et al. 2010, 2013; Person et al. 2010; Robroek et al. 2012; Rongen et al. 2014). A strategy to overcome the latter barrier might be participation in WHPPs during working hours. Nevertheless, our results indicate that when there is a lack of flexibility of work, e.g. not able to leave the workplace, a WHPP during working hours is a barrier. This emphasizes the importance of taking into account the resources, including private time and working schedules, of employees when implementing a WHPP. Various characteristics of individuals were identified as a barrier for participation in WHPPs in our study. These constructs might also be affected by organizational factors. For instance, a lack of energy and time might be explained by high (physical or mental) job demands or a lack of flexibility of work. Prioritizing family and friends over WHPPs has to do with work-life balance, which in turn might be related to the perceived workload as well. From other research it was observed that facilitators were social support from supervisors and co-workers and a positive attitude (Nöhammer et al. 2010; Rongen et al. 2014). These findings were in line with our data. Additionally, we found that a negative attitude or no belief in WHP hampered participation. According to Rongen et al., other factors that play an important role in whether an employee decides to participate or not are the preferences of an employee and the organizational environment (Rongen et al. 2014). These findings are supported by our findings and other literature (Nöhammer et al. 2010, 2013; Person et al. 2010).

### Strengths and limitations

The main strength of this study is the active participation of employees in collecting information about barriers and facilitators from their perspective. The peer-to-peer interview method is an innovative participatory approach (Tsui and Franzosa 2018). Advantages of the peer-to-peer interviews are enhanced research capacity and a positive change in behavior towards the study topic (Den Broeder et al. 2018; Project 2013). Further, interviewees are expected to answer more genuinely to their peers since a shared language and experiences make it easier to connect and create common ground and trust (Devotta et al. 2016; Elliott et al. 2001; Payne-Gifford et al. 2021; Quinney et al. 2016). On the other hand, despite the use of semi-structured interview cards, interviews cannot be redirected when they go off topic and in case of ambiguities the researcher cannot ask for clarification afterwards (Elliott et al. 2001; Payne-Gifford et al. 2021). However, this only occurred occasionally in this study. Moreover, none of the peer-interviewers had prior experiences in interviewing. To support the peer-interviewers as much as possible, a training for the peer-interviewers was provided, in which they practiced and received feedback on their interview skills. Nevertheless, closed questions were asked in a few interviews. For instance, the duration of one of the interviews was only three minutes. Hence, in future studies with peer-to-peer interviews extra guidance and support could be useful. For example, feedback can be provided after the first interviews, a helpdesk for questions can be set, or a researcher can be present during the first interviews. It should be considered that relevant information might be missed due to the lack of experience of the peer-interviewers. However, we expect this limitation to be partially mitigated by the high number of interviews that was carried out. Each peer-interviewer will focus on other topics, which overall is expected to lead to fairly complete information. Moreover, Devotta et al. argued that peer-interviews could even lead to richer qualitative data, due to a stronger connection between interviewer and interviewee (Devotta et al. 2016).

Due to the COVID-19 restrictions at the time of the study both the training and part of the interviews were online, which can be viewed as a limitation of our study. However, feedback about the online training from the peer-interviewers was positive and if there were any questions afterwards, they could easily reach the researchers. Some peer-interviewers used this opportunity. In online interviewing it might be more difficult to connect when body language is limited and poor network connections can interrupt the interviews (Moran and Caetano 2021). However, advantages and positive reactions on online interviewing have been reported, which indicates that online interviewing is an appropriate option to yield qualitative data (Gray et al. 2020; Moran and Caetano 2021). Reported advantages of online interviewing are accessibility and flexibility and participants are interviewed in their own chosen space. Despite the distance, there still is a more personal connection with the interviewer when compared to interviews over the phone.

Another strength of this study was the use of the CFIR, this framework can be used across various contexts, including implementation of WHPPs (Molin et al. 2021). Furthermore, it is designed to identify barriers and facilitators during the pre-implementation phase (Damschroder et al. 2009; Kirk et al. 2016). Since this is a study from the user perspective instead of the implementers’ perspective, we made small adaptations and added two constructs from the social ecological model. The framework suited the data and the purpose of this study, as only few additional codes emerged and no additional domains were necessary. Final strengths were the total number of interviews and the heterogenic group of employees that participated in this study. Various job types were represented, therefore, results can also be representative for other organizations.

Possible selection bias should be taken into account as all organizations recognize the importance of WHP. Moreover, employees that applied as peer-interviewers might be the employees who also consider WHP to be of importance. However, not all interviewees had the same idea about the importance of WHP. This might indicate that there is less selection bias on the level of interviewees. The fact that organizations were recruited through the network of the research team is not expected to influence the results of the peer-to-peer interviews, as the peer-interviewers and interviewees were not involved in the decision of the organization to participate in this study.

### Implications

To increase participation in future WHPPs it is important that employees have a positive attitude towards WHP, are aware of the WHP offer at the workplace and know what a healthy lifestyle entails. To achieve this, clear and active communication, tailored to the target group, about possibilities and the importance of WHP, is key (Nöhammer et al. 2010, 2013; Persson et al. 2013; WHO 2008). Hence, it is crucial that organizations actively inform their employees using a variety of communication channels, such as a personal approach, distribution of information by supervisors, e-mails and posters. Additionally, the facilitator social support should be deployed to positively affect the organizational environment. Thus, support from supervisors should be encouraged and enthusiastic employees should be appointed as ambassadors, to act as a role model for participation in WHPPs (Nöhammer et al. 2010; Person et al. 2010; Sigblad et al. 2020). Moreover, it is paramount for employers to be aware of the available resources and needs of employees. For this reason, employers should involve employees during the development and implementation of WHPPs (Nöhammer et al. 2010; Person et al. 2010; Robroek et al. 2012; WHO 2008). To intervene on barriers on the individual level, employers should critically review, and if necessary adjust, organizational factors, such as the perceived job demands of employees. Future research should assess whether considering these barriers and facilitators prior to implementation leads to an increase in participation.

## Conclusion

In conclusion, a supportive organizational culture and a positive individual attitude and knowledge seem necessary to increase participation of employees in WHPPs. This study showed that both individual factors and organizational factors play an important role in the participation of employees. Strategies to overcome barriers for participation will be incorporated in an implementation plan, to better implement the integrated WHPP. The effectiveness of the integrated approach, consisting of the catalogue and implementation plan, will be evaluated in a cluster randomized controlled trial. A process evaluation will provide more insight in the success of the implementation of the integrated WHPP. We recommend stakeholders, such as employers and occupational health and safety professionals, involved in the implementation of integrated WHPPs, to use this knowledge about barriers and facilitators for the implementation of future WHPPs.

## Data Availability

The datasets generated and analyzed during the current study are not publicly available due to some of the data being potentially identifiable. These data are available from the corresponding author on reasonable request.
